# Supplementary material for: A Switch from Cell-Associated to Soluble PDGF-B Protects against Atherosclerosis, despite Driving Extramedullary Hematopoiesis
Source: Cells. 2021 Jul 10;10(7):1746. doi: 10.3390/cells10071746 (PMC8308020; doi:10.3390/cells10071746)
Supplement: Supplementary file 1 [file cells-10-01746-s001.zip › cells-1277242-supplementary.pdf]

SUPPLEMENTARY MATERIALS

A switch from cell-associated to soluble PDGF-B protects against atherosclerosis, despite driving extramedullary hematopoiesis

Renée J.H.A. Tillie[1], Thomas L. Theelen[1], Kim van Kuijk[1], Lieve Temmerman[1], Jenny de Bruijn[1], Marion Gijbels[1-3], Christer Betsholtz[4], Erik A.L. Biessen[1,5], Judith C. Sluimer[1,6]

1. Department of Pathology, Cardiovascular Research Institute Maastricht (CARIM) Maastricht University Medical Center, Maastricht, the Netherlands
2. Department of Pathology, GROW-School for Oncology and Developmental Biology, Maastricht University Medical Center, Maastricht, the Netherlands
3. Department of Medical Biochemistry, Experimental Vascular Biology, Amsterdam UMC, University of Amsterdam, Amsterdam, the Netherlands
4. Department of Immunology, Genetics and Pathology, Uppsala University, Rudbeck Laboratory, Uppsala, Sweden
5. Institute for Molecular Cardiovascular Research (IMCAR), RWTH Aachen University, Aachen, Germany
6. BHF Centre for Cardiovascular Sciences (CVS), University of Edinburgh, Edinburgh, UK

Supplementary Materials and Methods

S1. Plasma cholesterol and triglyceride levels

Standard enzymatic techniques were used to assess plasma cholesterol (CHOD-PAP method – Cholesterol FS Ecoline no. 113009990314; DiaSys – Diagnostic Systems GmbH) and plasma triglycerides (FS5' Ecoline no. 157609990314; DiaSys – Diagnostic Systems GmbH) automated on the Cobas Fara centrifugal analyzer (Roche).

S2. Pro-inflammatory cytokines in plasma and BMDM conditioned medium

Cytokine levels in plasma and BMDM conditioned medium were assessed using a V-PLEX Proinflammatory Panel 1 Mouse Kit following manufacturer's protocol (K15048D-1, Meso Scale Diagnostics).

Supplementary Tables

Table S1: Immunohistochemical staining protocols of murine aortic root cryosections

	**αSMA**<br>Double staining with CD31	**CD31**<br>Double staining with αSMA	**PDGF-B**	**MOMA-2**
**Fixation**	Dry acetone		4% PFA in PBS	Dry acetone
**Permeabilization**	-		0.25% Triton-x100 in PBS	-
**Blocking**	0.3% H_2O_2 in methanol<br><br>Serum-free protein block (X0909, DAKO)		0.3% H_2O_2 in methanol	0.3% H_2O_2 in methanol<br><br>PBS 4% FCS and avidin block 1:5 (SP-2001, Vector)
**Primary antibody Cat. no and company**	F3777, Sigma FITC-conjugated	550274, BD	Ab23914, Abcam	Molecular Genetics department Maastricht University
**Primary antibody dilution and buffer**	1:300<br>TBS	1:25<br>TBS	1:700<br>TBT (TBS + 1% BSA + 0.1% Tween)	1:50<br>PBS, 4% FCS, biotin block 1:5 (SP-2001, Vector)
**Secondary antibody Cat. no and company**	Sheep anti-FITC-HRP, 11.426.346.910 Roche	Biotinylated rabbit anti-rat, BA-4001, Vector	Brightvision poly-HRP-anti-rabbit, DPVR-55-HRP, Immunologic	Biotinylated rabbit anti-rat, Molecular Genetics department Maastricht University
**Secondary antibody dilution and buffer**	1:300<br>TBS	1:200<br>TBS	-	1:300<br>PBS, 2% normal mouse serum (X0910, DAKO), 4% FCS
**Tertiary step/antibody Cat. no and company**	-	ABC-AP kit (AK-5000, Vector)	-	ABC-HRP kit (PK-4000, Vector)
**Stain development**	Diaminobenzidine kit (K346811-2, Agilent)	Vector Blue substrate kit, alkaline phosphatase (SK-5300, Vector)	Diaminobenzidine kit (K346811-2, Agilent)	AEC kit (2% buffer, 3% AEC, 2% H_2O_2 in milliQ, K3461, DAKO)

FCS; fetal calf serum, PBS; phosphate-buffered saline, PFA; paraformaldehyde, TBS; tris-buffered saline.

Table S2: Primer sets used for genotype confirmation through PCR and electrophoresis

Gene	Forward primer (5'- 3')	Reverse primer (5'- 3')
Pdgfb^{WT}	CATGCTGCCTTGTAATCCGTTC	CGGCGGATTCTCACCGT
Pdgfb^{ret}	CTCGGGTGACCATTCGGTAA	TCTAAGTCACAGCCAGGGAGT AGC

Table S3: Primer sets used for qPCR

Gene	Forward primer (5'- 3')	Reverse primer (5'- 3')
18s rRNA	GTAACCCGTTGAACCCCATT	CCATCCAATCGGTAGTAGCG
Pdgfb	CGGTCCAGGTGAGAAAGATTG	CGTCTTGGCTCGCTGCTC
Pdgfra	AGAGAGAATCGGCCCCAGTG	CCATAGCTCCTGAGACCCGC
Pdgrb	GGCCTTAGTGGTCCTTACCG	GCACAGGGTCCACGTAGATG

Table S4: Antibodies used for flow cytometry

Antibody	Company	Catalog number	Dilution used
CD16/32	eBioscience	14-0161-82	1:100
CD45 PerCP	Biolegend	103130	1:100
CD3 eFluor 450	eBioscience	48-0032-82	1:100
NK-1.1 PE	BD Pharmingen	557391	1:100
Ly6G APC-Cy7	BD Pharmingen	560600	1:100
CD11b PE-Cy7	BD Pharmingen	552850	1:300
Ly-6C APC	Miltenyi Biotec	130-102-341	1:10
CD4 APC-H7	BD Pharmingen	560181	1:100
CD8 V500	BD Horizon	560776	1:200
CD8 FITC	eBioscience	11-0081-85	1:50
B220 V500	BD Horizon	561226	1:50
Sca-1 PerCP-Cyanine 5.5	eBioscience	45-5981-82	1:1000
c-Kit APC-eFluor780	eBioscience	47-1171-82	1:100
CD34 eFluor450	eBioscience	48-0341-82	1:50
CD16/32 PE-Cy7	eBioscience	25-0161-82	1:1000
B220 PE	BD Pharmingen	553089	1:100
CD3 PE	eBioscience	12-0031-82	1:800
CD11b PE	eBioscience	12-0112-82	1:800
Ly-6G PE	BD Pharmingen	551461	1:100
NK-1.1 PE	BD Pharmingen	557391	1:100
TER-119 PE	eBioscience	12-5921-82	1:200

Supplementary Figures

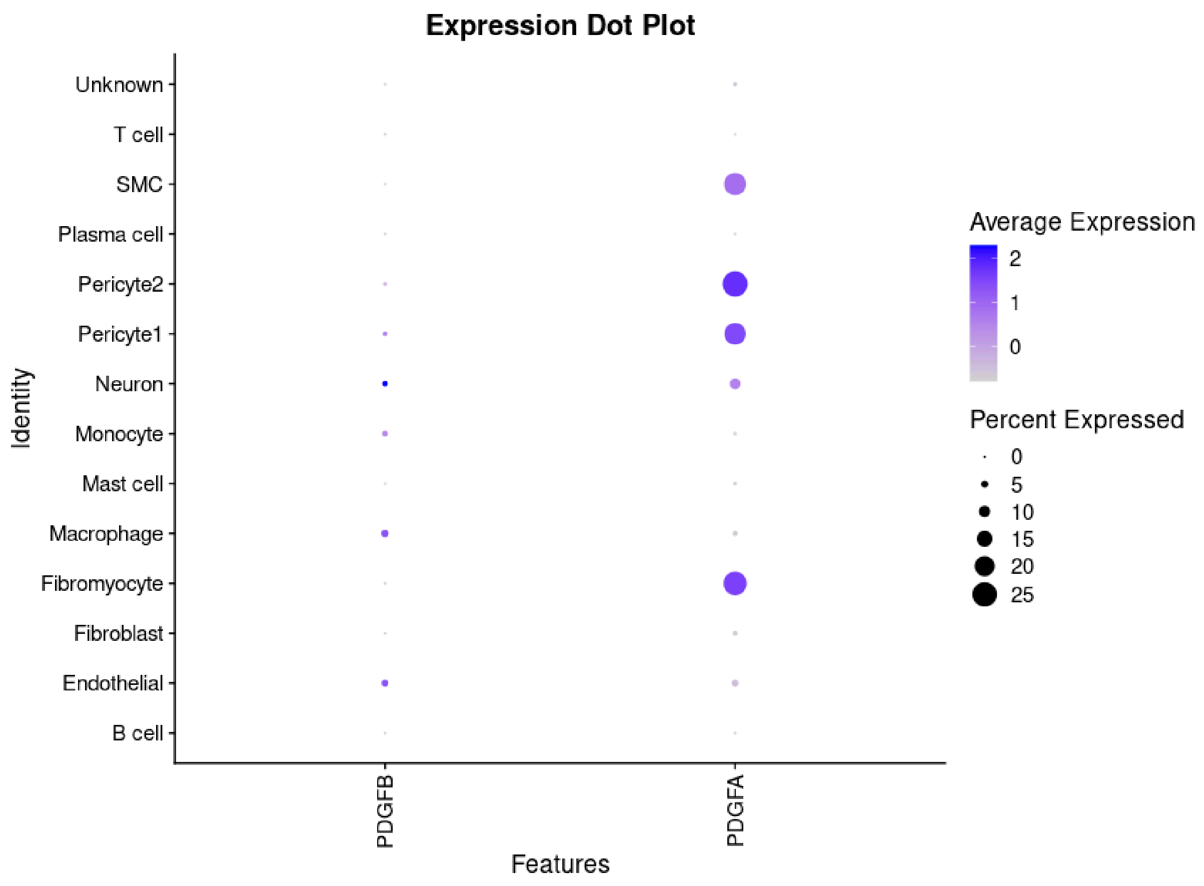

Figure S1: *PDGFB and PDGFA* expression in human atherosclerosis
Dot plot of *PDGFB* and *PDGFA* expression in single cell populations of human atherosclerotic coronary arteries (Wirka et al. [21]). SMC; smooth muscle cell.

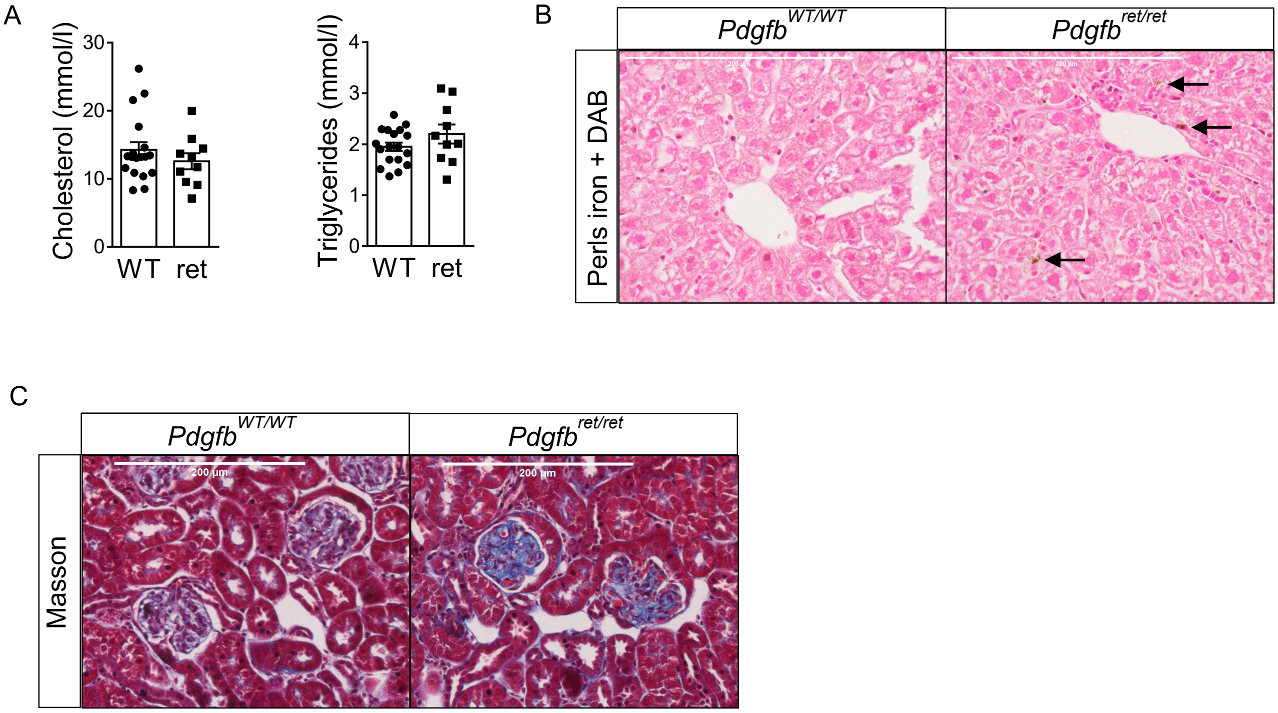

Figure S2: Similar cholesterol and triglyceride levels in *Pdgfb*$^{WT/WT}$ and *Pdgfb*$^{ret/ret}$ plasma and affected *Pdgfb*$^{ret/ret}$ liver and kidney

(**A**) Cholesterol and triglyceride levels in *Pdgfb*$^{WT/WT}$(*n*=18) and *Pdgfb*$^{ret/ret}$(*n*=10) plasma. (**B**) Representative photomicrographs of Perls iron staining combined with DAB in *Pdgfb*$^{WT/WT}$ and *Pdgfb*$^{ret/ret}$ liver. (**C**) Representative photomicrographs of Masson staining in *Pdgfb*$^{WT/WT}$ and *Pdgfb*$^{ret/ret}$ kidney, in which ECM is stained blue. Scale bars 200 µm. Data were tested for normality (Shapiro-Wilk) and equal variances (F-test). Variables that did not pass, were analyzed using Mann-Whitney U test. Variables that did pass, were analyzed using Student's t-test.

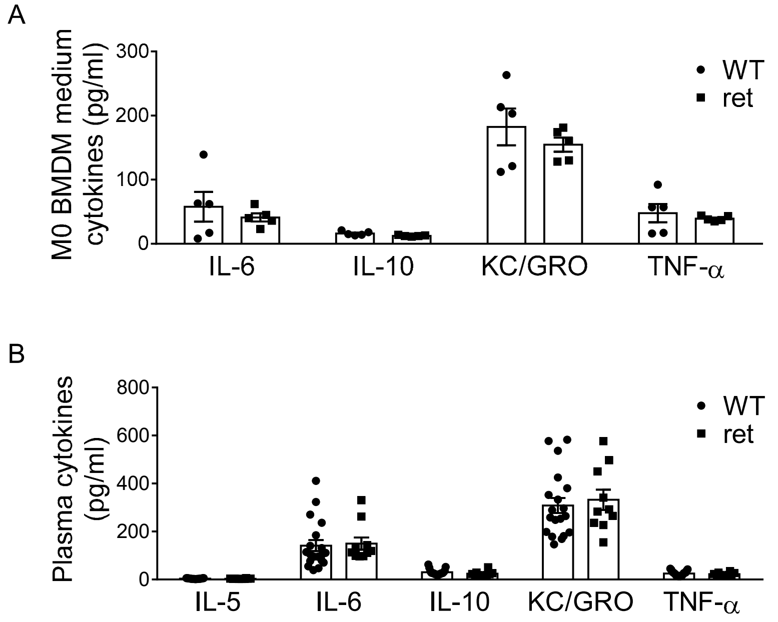

Figure S3: Pro-inflammatory cytokine levels in plasma and BMDM conditioned medium unaffected. (**A**) Levels of interleukin 6 (IL-6), IL-10, keratinocyte-derived chemokine/growth-related oncogene (KC/GRO or CXCL1) and tumor necrosis factor-α (TNFα) in BMDM-derived medium (*n*=5). IFN-γ, IL-1β, IL-5, IL-12 p70, IL-2 and IL-4 levels were undetectable. (**B**) Levels of IL-5, IL-6, IL-10, KC/GRO and TNF-α in $Pdgfb^{WT/WT}$(*n*=19) and $Pdgfb^{ret/ret}$(*n*=10) plasma. IFN-γ, IL-1β, IL-12 p70, IL-2 and IL-4 levels were undetectable. Graphs represent mean±SEM. Data were analyzed using two-way ANOVA.

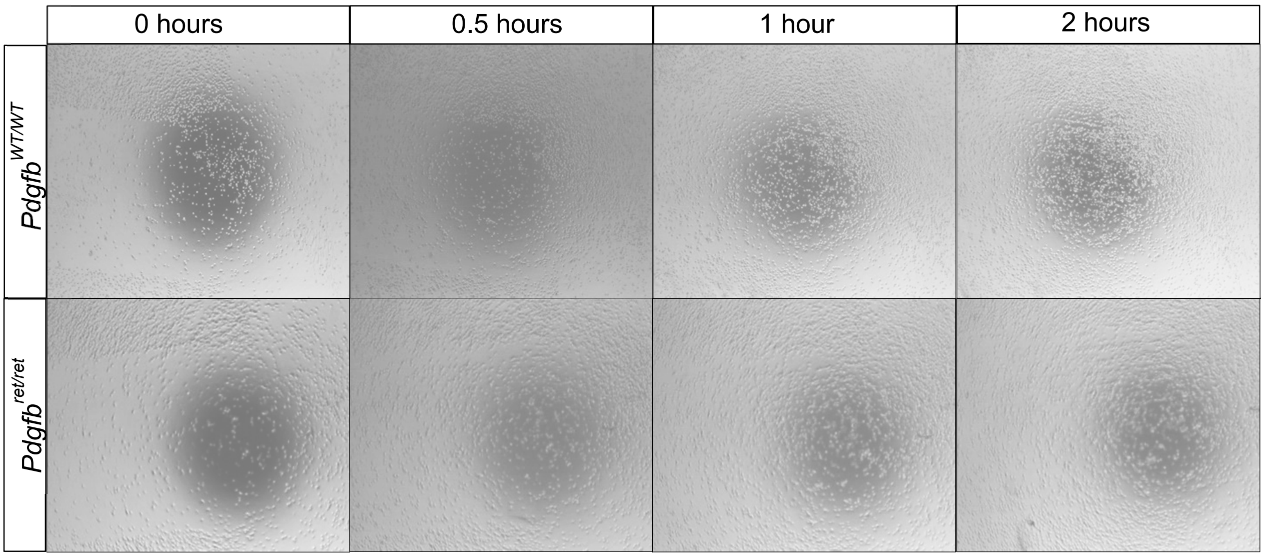

Figure S4: Pictures of BMDM scratch assay to assess migration *in vitro*.
Representative pictures of *Pdgfb*$^{WT/WT}$ and *Pdgfb*$^{ret/ret}$ BMDM migration over time (t=0, 0.5 hours, 1 hour and 2 hours) after scratch infliction.

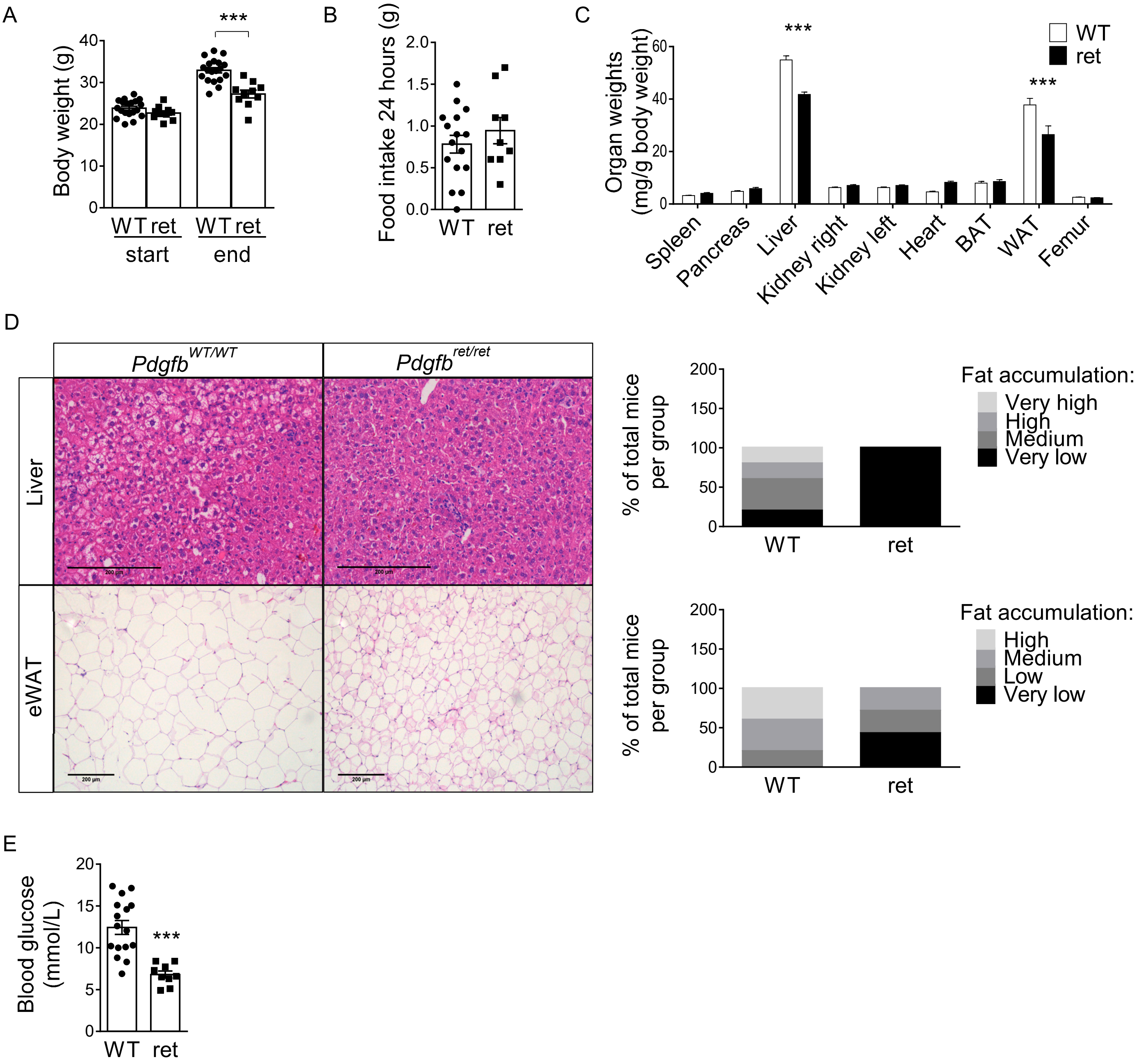

Figure S5: Decreased body weight gain and fat accumulation in *Pdgfb*$^{ret/ret}$ liver and epididymal white adipose tissue (eWAT). (**A**) *Pdgfb*$^{WT/WT}$(n=19) and *Pdgfb*$^{ret/ret}$(n=10) body weight before and after 10 weeks of high cholesterol diet (HCD) (**B**) 24-hour food intake as assessed in metabolic cages, in the 9th week of HCD. (**C**) Organ weights after 10 weeks of HCD, relative to body weight. (**D**) Fat accumulation as assessed by visual analogue scores ranging from very low to very high fat accumulation in HE-stained *Pdgfb*$^{WT/WT}$(n=5) and *Pdgfb*$^{ret/ret}$(n=7) liver and eWAT, with corresponding photomicrographs. (**E**) Blood glucose levels after 10 weeks of HCD. Results shown in B, C and E were obtained using 16 *Pdgfb*$^{WT/WT}$ and 9 *Pdgfb*$^{ret/ret}$ mice. Scale bars 200 µm. Graphs represent mean±SEM. ***$p<0.001$. Data were tested for normality (Shapiro-Wilk) and equal variances (F-test). Variables that did not pass, were analyzed using Mann-Whitney U test. Variables that did pass, were analyzed using Student's t-test. Data in A and C were analyzed using two-way ANOVA, including Bonferroni's multiple comparisons test.

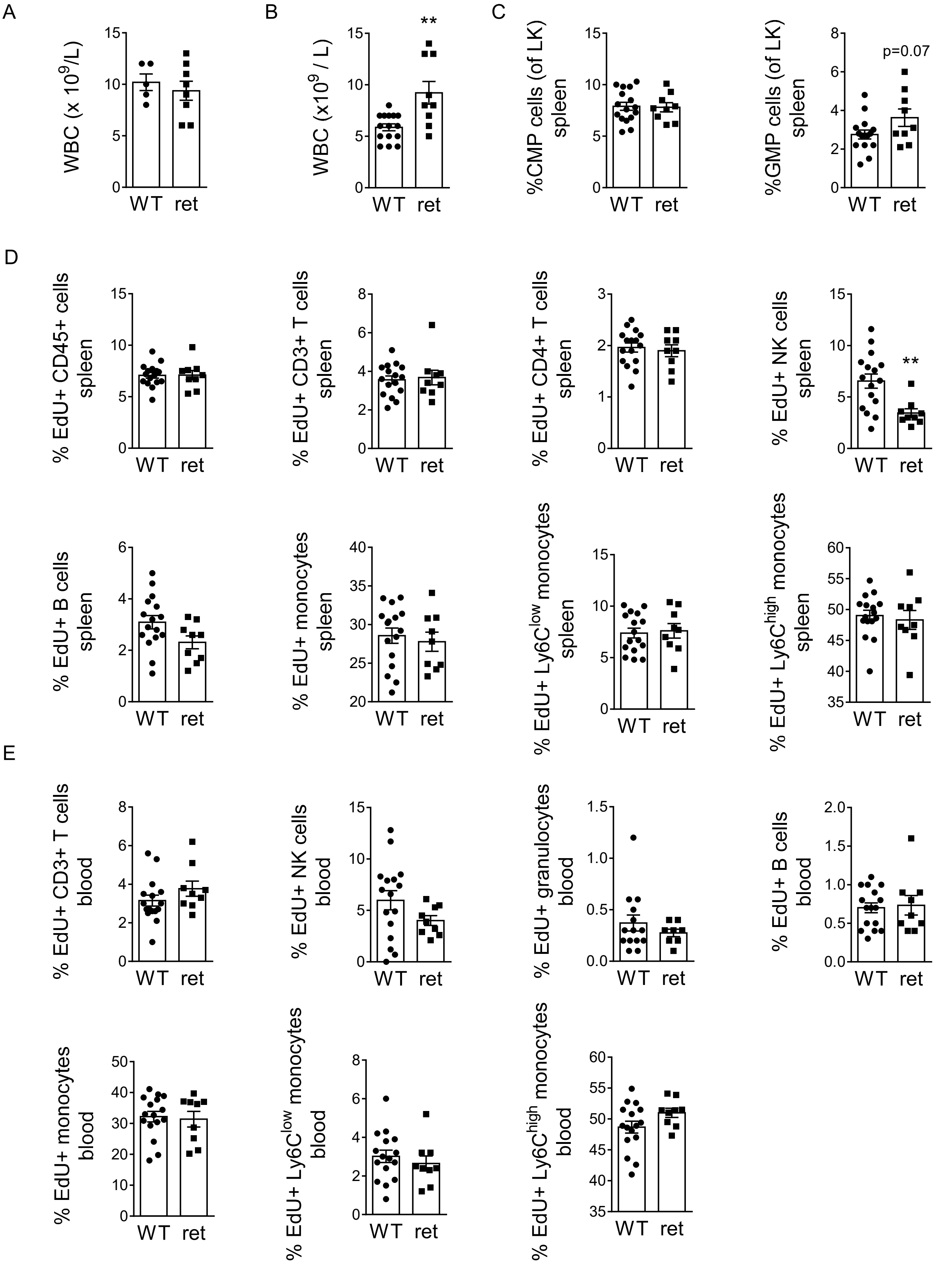
A
WBC (x 10^9/L)
WT ret
B
**
WBC (x10^9 / L)
WT ret
C
%CMP cells (of LK) spleen
WT ret
%GMP cells (of LK) spleen
p=0.07
WT ret
D
% EdU+ CD45+ cells spleen
% EdU+ CD3+ T cells spleen
% EdU+ CD4+ T cells spleen
% EdU+ NK cells spleen
**
% EdU+ B cells spleen
% EdU+ monocytes spleen
% EdU+ Ly6C^low monocytes spleen
% EdU+ Ly6C^high monocytes spleen
E
% EdU+ CD3+ T cells blood
% EdU+ NK cells blood
% EdU+ granulocytes blood
% EdU+ B cells blood
% EdU+ monocytes blood
% EdU+ Ly6C^low monocytes blood
% EdU+ Ly6C^high monocytes blood
WT ret

Figure S6: *Pdgfb$^{ret/ret}$* leukocytosis confirmation in second mouse experiment after HCD and percentages of EdU positive leukocytes in blood and spleen. (**A**) White blood cell (WBC) counts in blood from *Pdgfb$^{WT/WT}$*(*n*=5) and *Pdgfb$^{ret/ret}$*(*n*=8) mice on standard laboratory diet. (**B**) WBC counts in *Pdgfb$^{WT/WT}$*(*n*=15) and *Pdgfb$^{ret/ret}$*(*n*=9) blood after 10 weeks HCD in the second mouse experiment. (**C**) Percentage progenitor cells of lineage^{-}c-Kit^{+} in *Pdgfb$^{WT/WT}$*(*n*=15-16) and *Pdgfb$^{ret/ret}$*(*n*=9) spleen. CMP = common myeloid progenitor, GMP = granulocyte monocyte progenitor. (**D**) Percentage of EdU positive leukocytes in *Pdgfb$^{WT/WT}$*(*n*=14-16) and *Pdgfb$^{ret/ret}$*(*n*=9) spleen and (**E**) blood. Graphs represent mean±SEM. **p<0.01. Data were tested for normality (Shapiro-Wilk) and equal variances (F-test). Variables that did not pass, were analyzed using Mann-Whitney U test. Variables that did pass, were analyzed using Student's t-test.
